# Next-Generation Sequencing Analysis of 3 Uterine Adenosarcomas with Heterogeneously Differentiated Genomic Mutations

**DOI:** 10.1155/2023/7436368

**Published:** 2023-09-28

**Authors:** Yao Li, Xue Meng, Yuqing Luo, Xiang Huang, Shuai Luo, Jinjing Wang

**Affiliations:** Department of Pathology, Affiliated Hospital of Zunyi Medical University, Zunyi, Guizhou, China

## Abstract

Uterine adenosarcoma (UA) is an uncommon mixed tumor containing a benign to at most mildly atypical epithelial component and a sarcoma-like stroma, usually a low-grade, stromal component, with rare heterogeneous elements. Currently, tumor etiology is largely unknown. To better understand the gene mutations in UA, next-generation sequencing (NGS) technology analysis was performed. This study showed that two low-grade UAs with heterologous components had ATRX gene frameshift mutation, and one patient had a MED12 missense mutation. Copy number amplification genes were mainly observed on chromosome 12q^13–15^. In this study, PIK3/AKT/PTEN pathway mutations were found to be common in adenosarcoma. In addition, a rare BCORL1-PRR14L fusion mutation was also identified. These findings provide a basis for future research into these molecular changes in tumorigenesis and targeted therapy.

## 1. Introduction

Uterine adenosarcoma (UA) is an uncommon mixed tumor containing a benign to at most mildly atypical epithelial component and a sarcoma-like stroma and accounts for approximately 5% of uterine sarcomas [1]. The epithelial component is Müllerian-derived, mainly endometrioid epithelium and a few visible tubal epithelium and squamous epithelium; the glandular epithelium may be accompanied by different atypical degrees. The stroma is generally a low-grade uterine sarcoma, mostly endometrial stromal sarcoma [2], which may contain heterologous components, such as the skeletal muscle, cartilage, and smooth muscle, among others. However, adenosarcoma with heterologous components is rare [3], and the molecular mechanism is unclear. Two of the three UAs reported in this study had heterologous components, and the molecular changes were detected by next-generation sequencing (NGS) to understand the pathogenesis and identify diagnostic or prognostic biomarkers.

## 2. Materials and Methods

### 2.1. Case Selection

The specimen-related and existing clinical data of three patients with pathologically diagnosed UA treated in our hospital from 2017 to 2022 were obtained.

### 2.2. Next-Generation Sequencing (NGS)

DNA was extracted from tissue samples based on the instructions in the QIAGEN DNA FFPE extraction kit (QIAGEN, Valencia, CA, USA). The quality control samples were arranged for the construction of the library. The 481 gene probe was used for targeted enrichment of the DNA samples, and the target enrichment library was sequenced on the NovaSeq 6000 platform (Illumina). Finally, comprehensive information regarding genetic mutations, such as point mutation, insertion and deletion mutations, and gene copy number change, was obtained from the sequencing data using the genome analysis toolkit.

## 3. Results

### 3.1. Clinicopathological Features

The clinicopathological characteristics of the patients are summarised in Table 1. The age of the three patients ranged from 57 to 76 years (average, 67 years). The sites of occurrence of the tumors were the uterine fundus, corpus, and cervix. Three patients were admitted because of abnormal vaginal bleeding. All three patients underwent abdominal hysterectomy and bilateral adnexectomy. The postoperative pathological macroscopic image revealed that the uterus was enlarged and single or multiple polypoid masses protruded into the uterine cavity or cervical canal. The average diameter of the tumors was 6.9 cm (range, 2.8–14 cm). The surface of the tumors was smooth, and the cut surface was fish flesh-like, with grey red color and tender texture. The tumors had a saccular shape with local bleeding and necrosis.

**Figure 1:**
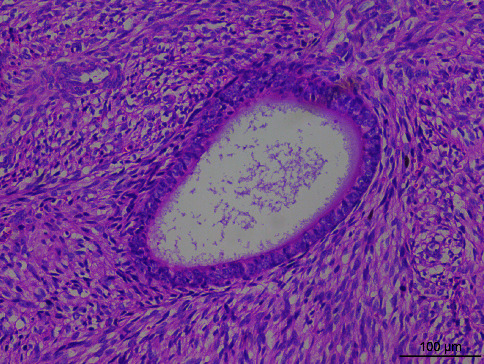
Local tumor cells were concentrated around the gland, forming a cuff structure around the gland (H&E ×200).

**Figure 2:**
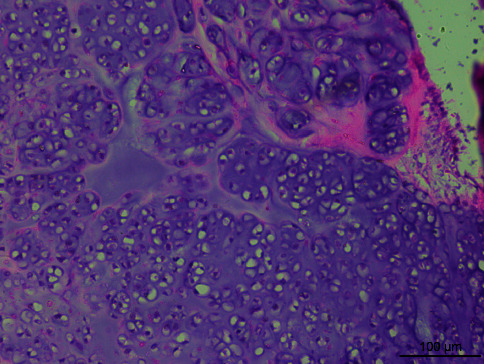
Microphotograph showing heterogenic differentiation with a mature cartilage (HE ×400).

**Figure 3:**
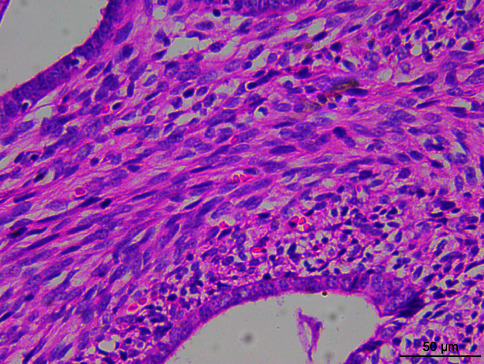
Fusiform nuclei with mild to moderate atypia, and local mitotic images are easy to see (HE ×200).

Microscopically, the tumor tissue was composed of endometrioid glands and proliferative spindle cells. A large number of tumor cells were diffused and distributed in a woven and cord-like arrangement, and local tumor cells were concentrated around the gland, forming a cuff structure around the gland (Figure 1). Fusiform nuclei with mild to moderate atypia and local mitotic images are easy to see (Figure 2). One case had heterogeneous differentiation of tumor cells into chondrocytes (Figure 3), and another case had smooth muscle differentiation. No definite tumor thrombus was found in the vasculature.

Immunohistochemical examination showed positive expression of CK7 and EMA in glandular epithelium. CD10 and vimentin were positive in the interstitium; desmin, SMA, and PR were partially positive in one patient, and ER was partially positive in two patients. P53, inhibin, caldesmon, myogenin, and MyoD1 were negative in all three patients, and the positivity rate of Ki67 was 5%–15%. Combined with the histopathological and immunohistochemical findings of the patients, adenosarcoma of the uterus was diagnosed. None of the patients received radiotherapy or chemotherapy after surgery. The follow-up time ranged from 2 months to 36 months. One patient had vaginal recurrence after 17 months.

### 3.2. Overview of Genomic Changes

**Figure 4:**
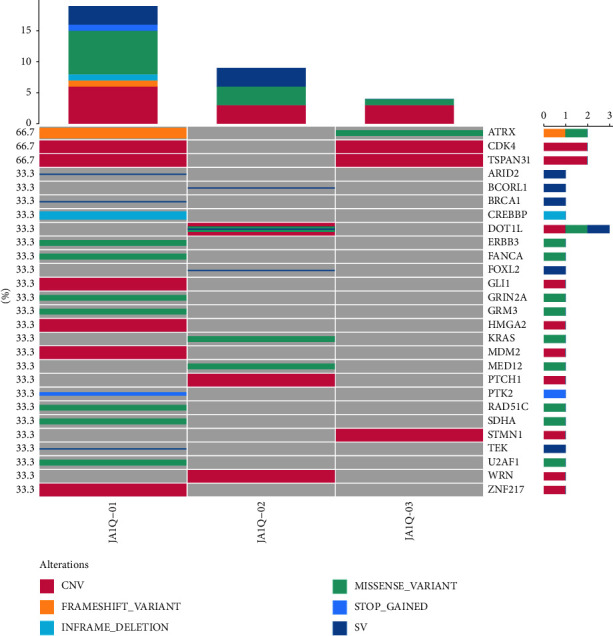
Target validation of the gene mutation types and distribution of 27 genes in 3 UAs by next-generation sequencing (NGS). Rows represent individual genes and columns represent individual tumors. Mutated genes are sorted according to frequency in this cohort. Colors indicate the mutation type detected in each tumor.

**Figure 5:**
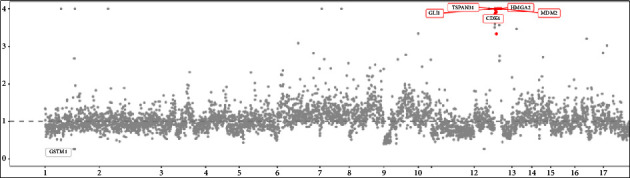
Copy number variation data for chromosome 12 in CNV analysis, with the plot of copy number variation by chromosome (color-coded). The vertical axis is the ratio of the number of reads for this specimen versus a panel of normals in log base 2 scale. A value of 0 denotes no difference from normal (diploid). The horizontal axis shows the chromosomes.

**Figure 6:**
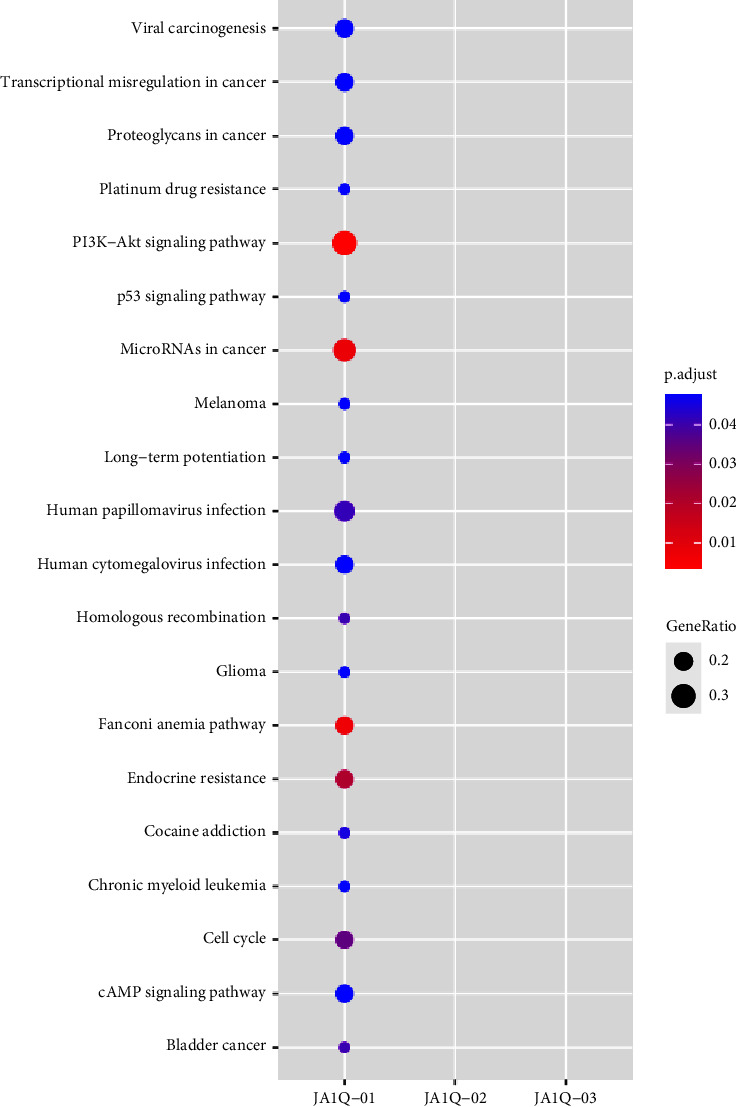
KEGG pathway analysis of oncogenes/tumor suppressor genes altered in UAS; the redder the circle color, the more significant the enrichment, and the larger the circle, the more enriched the genes.

The genomic variants identified in the patients are summarised in Figure 4, including genes with pathogenic mutations (ATRX, PTK2, PTCH1, and KRAS) and novel variants of unknown significance (FANCA, CREBBP, FOXL2, WRN, and DOT1L). Two patients with heterologous differentiation had the ATRX gene frameshift mutation (p.S788, case 1; p.P2141S, case 3). The other type was missense mutations in DOT1L (p.E262K, p.K401N, patient 2), KRAS (12p12.1, patient 2), and MED12 (p.Q2097L, case 2). The present study found that copy number amplification of genes (CDK4, GLI1, HMGA2, MDM2, and TSPAN31) mainly occurred on chromosome 12q^13–15^ (Figure 5). In addition, this study found gene fusion mutations, including PLEKHA7-BRCA1, PPFIA2-ARID2, RAB22A-TEK, DOT1L-ZNF57, and BCORL1-PRR14L fusions. Furthermore, we found that PIK3/AKT/PTEN pathway mutations were the most common in adenosarcomas through the Kyoto Encyclopedia of Genes and Genomes (KEGG) enrichment analysis (Figure 6).

## 4. Discussion

UA, also known as Müllerian adenosarcoma [3], was defined by the World Health Organization as a mixed epithelial and mesenchymal tumor [4]. UA occurs most frequently in the uterine body, followed by the cervix, and about 1/4 showed other heterologous interleaflet components, with striated muscle differentiation being the most common [5, 6]. In this study, it is rare that one patient had tumors in the uterine cavity and cervix simultaneously. The age of onset of UA ranges from 10 to 94 years (median age, above 50 years) [7] and is more common in postmenopausal women. Overall, UA has a better prognosis than other uterine sarcomas and carcinosarcomas [8, 9]. However, Heveder et al. [10–12] suggested a 50% reduction in survival in UA patients with adverse prognostic factors. Although surgery is the major treatment [13], the therapeutic effect of chemotherapy in UA has been reported in the literature [14].

Some commonalities were identified between copy number variations (CNVs) and mutations. A study has shown that UA and its variants are genetically heterogeneous with frequent CNVs in SO [15]. Meanwhile, MDM2, CDK4, HMGA2, and GLI1 gene amplifications are common molecular events in Müllerian adenosarcoma, often seen in patients with SO [16]. Consistently, in this study, gene amplification was seen in recurrent UAs and UAs with heterologous differentiation without SO.

Furthermore, the MED12 gene is closely related to uterine leiomyoma [17]. The 70% of uterine leiomyomas have point mutations in the MED12 gene, and all relevant point mutations are concentrated in exon 2 [17]. MED12 mutations alter the functioning of the MED12 protein, thus disrupting normal cell signalling and repair regulation of cell growth and other functions, resulting in uncontrolled cell growth and tumorigenesis [17]. In contrast, this study has detected a missense mutation in exon 43 of the MED12 gene, p.Q2097L, resulting in the change of the 6290th base from A to T and the change of the 2097^th^ amino acid from glutamine to leucine. However, the clinical significance of this mutation is unclear. Nevertheless, if the protein functions abnormally, it may affect downstream signalling pathways and be involved in tumorigenesis and progression of cancer.

Moreover, ATRX (located on chromosome Xq21.1) encodes a chromatin remodelling protein which is thought to be important in regulating DNA methylation and telomere stability [18]. Howitt et al. [16] reported that ATRX mutations were present in 50% of UAs with SO (including one case with distant metastasis), while ATRX mutations were not present in UAs without SO, suggesting that ATRX may be a poor prognostic feature of MA [19, 20]. This is inconsistent with findings of this study, in which ATRX mutations were occurred in low-grade UAs with heterologous components but not with SO. Therefore, there requires further studies to confirm these findings.

In addition, structural variation (SV) is relatively low in UAs. This study reported a fusion mutation in the BCORL1-PRR14L gene. Meanwhile, the diagnosis of BCOR overexpressing uterine sarcomas and high-grade endometrial mesenchymal sarcomas carrying these mutations is suggested by identifying ZC3H7B-BCOR fusion mutations or BCOR-ITD [21]. However, the clinical significance of this mutation in UAs is currently unclear. If the protein functions abnormally, it may affect downstream signalling pathways involved in tumor development and progression. Analysis of these gene fusions may provide clues to further understanding of the genetic mechanisms involved in UA tumorigenesis.

PIK3/AKT/PTEN pathway mutations are most common in adenosarcoma [16]. The findings of this study consistently indicated that targeting this pathway may be a potential therapeutic target in UA treatment. Moreover, surgical resection is the main treatment for UA, and hysterectomy with bilateral adnexectomy is the basic surgical method [22]. Previous studies have reported that the recurrence rate of UA is 14.3–46%, and the local recurrence rate is higher than the distant recurrence rate [8, 23]. The high-risk factors might affect the prognosis of adenosarcoma [24] and therefore individualized treatment should be considered. In terms of adjuvant therapy, it has been shown that adenosarcoma recurrence with or without sarcoma overgrowth responds to the treatment regimen of ifosfamide or doxorubicin [25]. However, radiotherapy does not benefit the overall survival of patients, and there is insufficient evidence regarding the benefits of chemotherapy and hormone therapy [25]. Further studies are needed to determine the most effective adjuvant therapy.

## 5. Conclusion

In conclusion, UA is a rare uterine sarcoma. This study showed that the ATRX gene was mutated in low-grade UA with a heterozygous component, possibly having important prognostic implications. Meanwhile, molecular evaluation of mutations in the BCOR and BCORL1 genes in the diagnosis of uterine sarcomas overexpressing BCOR is recommended to differentiate high-grade endometrial mesenchymal sarcomas with BCOR fusions from rare adenosarcomas with BCORL1 gene rearrangements and BCORL1-PRR14L fusions, potentially broadening the genetic spectrum of adenosarcomas. Future studies should be conducted on a larger sample, along with a detailed study of the mutated genes.

## Figures and Tables

**Table 1:** Clinical features of uterus adenosarcoma.

Case no.	Age (years)	Symptoms	Tumour size (cm)	Stage	SO	Microscopy (hd)	Surgery	Adjuvant therapy	Time to recurrence (months)	Site(s) of recurrence	follow-up(months)
1	57	Abnormal vaginal bleeding	4	IA	(−)	Cartilage	TAH, BSO	None	N/A	N/A	2
2	76	Abnormal vaginal bleeding	14	IA	(−)	N/A	TAH, BSO	None	N/A	N/A	26
3	68	Abnormal vaginal bleeding	2.8	IA	(−)	Smooth muscle	TAH, BSO	None	17	Vagina	36

N/A = not applicable; TAH = total abdominal hysterectomy; BSO = bilateral salpingo-oophorectomy; SO = sarcomatous overgrowth.

## Data Availability

The data presented in this study are available on request from the corresponding author.

## Abbreviations

CNVs: Copy number variations
ER: Estrogen receptor
H&E: Hematoxylin and eosin
IHC: Immunohistochemistry
IRB: Institutional Review Board
NGS: Next-generation sequencing
PR: Progesterone receptor
SO: Sarcomatous overgrowth
SV: Structural variation
UA: Uterine adenosarcoma.

## Supplementary Materials

Supplementary Materials Supplementary Table 1: the detailed information about ATRX gene.
